# Protein expression of TTF1 and cMYC define distinct molecular subgroups of small cell lung cancer with unique vulnerabilities to aurora kinase inhibition, DLL3 targeting, and other targeted therapies

**DOI:** 10.18632/oncotarget.20621

**Published:** 2017-09-01

**Authors:** Robert J. Cardnell, Lerong Li, Triparna Sen, Rasha Bara, Pan Tong, Junya Fujimoto, Abbie S. Ireland, Matthew R. Guthrie, Sheila Bheddah, Upasana Banerjee, Nene N. Kalu, You-Hong Fan, Scott J. Dylla, Faye M. Johnson, Ignacio I. Wistuba, Trudy G. Oliver, John V. Heymach, Bonnie S. Glisson, Jing Wang, Lauren A. Byers

**Affiliations:** ^1^ Department of Thoracic/Head and Neck Medical Oncology, The University of Texas MD Anderson Cancer Center, Houston, TX, USA; ^2^ Department of Bioinformatics and Computational Biology, The University of Texas MD Anderson Cancer Center, Houston, TX, USA; ^3^ Department of Molecular Translational Pathology, The University of Texas MD Anderson Cancer Center, Houston, TX, USA; ^4^ Department of Oncological Sciences, University of Utah, Huntsman Cancer Institute, Salt Lake City, UT, USA; ^5^ AbbVie StemCentrx, LLC, San Franciso, CA, USA; ^6^ The University of Texas Graduate School of Biomedical Sciences, Houston, TX, USA

**Keywords:** Alisertib, DLL3, rovalpituzumab tesirine, SCLC, TTF1

## Abstract

Small cell lung cancer (SCLC) is a recalcitrant cancer for which no new treatments have been approved in over 30 years. While molecular subtyping now guides treatment selection for patients with non-small cell lung cancer and other cancers, SCLC is still treated as a single disease entity. Using model-based clustering, we found two major proteomic subtypes of SCLC characterized by either high thyroid transcription factor-1 (TTF1)/low cMYC protein expression or high cMYC/low TTF1. Applying “drug target constellation” (DTECT) mapping, we further show that protein levels of TTF1 and cMYC predict response to targeted therapies including aurora kinase, Bcl2, and HSP90 inhibitors. Levels of TTF1 and DLL3 were also highly correlated in preclinical models and patient tumors. TTF1 (used in the diagnosis lung cancer) could therefore be used as a surrogate of DLL3 expression to identify patients who may respond to the DLL3 antibody-drug conjugate rovalpituzumab tesirine. These findings suggest that TTF1, cMYC or other protein markers identified here could be used to identify subgroups of SCLC patients who may respond preferentially to several emerging targeted therapies.

## INTRODUCTION

Small cell lung cancer (SCLC) is the most aggressive form of lung cancer, with a 5-year survival rate of less than 6% [1]. Although the majority of SCLC patients respond to chemotherapy and radiation initially, relapse is nearly universal among extensive stage patients and current treatment options for relapsed SCLC are largely ineffective [2]. A major barrier to the lack of progress in SCLC is an incomplete understanding of heterogeneity between patients and the absence of biomarkers that could guide selection of personalized therapeutic strategies. This is in contrast to non-small cell lung cancer (NSCLC) where a growing number of biomarker-defined subsets have been discovered that predict response to targeted or immunotherapies. These biomarker defined subsets include mutations in *EGFR*, *BRAF*, *MET*; fusions in *ALK*, *ROS1*, *RET*; and now PD-L1 protein expression levels. The development of biomarkers that can define subsets of SCLC patients with specific therapeutic vulnerabilities is urgently needed and will increase the likelihood of targeted therapies being successfully developed.

Currently, there is a single standard of care applied across all SCLC patients. However, in the clinic, we see a range of responses to chemotherapy as well as to targeted agents being tested in clinical trials in unselected SCLC populations, suggesting benefit in a small (but undefined) subset of patients. We previously demonstrated the potential of proteomic profiling to identify novel therapeutic targets in SCLC such as the DNA repair proteins PARP1 and CHK1 based on differences in target expression and pathway activation between SCLC and NSCLC [3, 4]. We further used proteomic data to identify predictive biomarkers for targeted therapies in SCLC models [4, 5] leading to the clinical development of new combination strategies to overcome drug resistance [6, 7].

In previous studies using reverse phase protein arrays (RPPA) to measure the expression of total and phosphorylated proteins in SCLC models, we observed heterogeneous protein expression profiles among preclinical SCLC models, as well as a wide range of *in vitro* drug sensitivities. Given the rapid evolution of lung cancer treatment towards a personalized, biomarker-guided approach using both genomic and proteomic markers, the finding that subgroups of SCLC respond differently to specific targeted approaches is not unexpected, but is incompletely understood. We propose that further categorization of SCLC based on protein expression could help define clinically-relevant molecular subsets of SCLC that could ultimately inform the clinical development of emerging therapies for SCLC such as those targeting DLL3, aurora kinase, Bcl2, and immune checkpoints [8].

Recent studies in a number of cancer types have used molecular data to identify subsets of disease that have different prognoses and responses to therapies [9-13]. While RPPA measures a discrete number of targets enriched for druggable and oncologically important pathways (typically around 200 total/phosphorylated proteins), it offers significant advantages over other approaches. For example, proteomics, unlike DNA or RNA-based profiling, directly measures pathway activation and candidate target expression (e.g., the protein “target” itself). Furthermore, protein biomarkers-particular those that can be assayed by immunohistochemistry (IHC)-have the potential for rapid translation into the clinic, as illustrated by the clinical use of PD-L1 IHC in NSCLC [14], and MET IHC in breast cancer [15]. Finally, in contrast to NSCLC where mutational profiling has defined targetable driver genes (e.g., *EGFR*), no such actionable single-gene drivers have been identified to date in SCLC. As such, the identification of proteomically defined subsets of SCLC has great potential to identify readily actionable markers to select patients for treatments undergoing clinical development or new targets and therapeutic susceptibilities in defined SCLC populations.

In this study, we performed proteomic profiling of a large panel of SCLC cell lines and then applied a model-based clustering approach to identify distinct subtypes of SCLC. Differentially expressed total proteins driving subtype membership were then further validated in preclinical models and two independent clinical cohorts [16, 17]. Having defined two groups of SCLC, we further explored differences between these groups by analyzing expression of specific proteins and/or genes, and response to potential therapeutic agents. Using a novel drug mapping approach, we created “drug target constellation” maps - DTECT maps - which identified drugs with differential sensitivity based on marker expression, and linked these drugs based on their primary, secondary and tertiary targets. Through DTECT mapping, low thyroid transcription factor-1 (TTF1) and high cMYC were confirmed as biomarkers of response to several drugs in development for SCLC, including the aurora kinase inhibitor alisertib (MLN8237). As TTF1 and cMYC protein can be assessed by IHC assays routinely used in clinical pathology labs, they represent highly translational markers for agents in development for SCLC. Potential applications of these biomarkers include the use of TTF1 to predict DLL3 levels (associated with response to the antibody-drug conjugate rovalpituzumab tesirine) and of cMYC to predict benefit from alisertib.

## RESULTS

### Clustering identifies two proteomically defined subsets of SCLC

**Figure 1:**
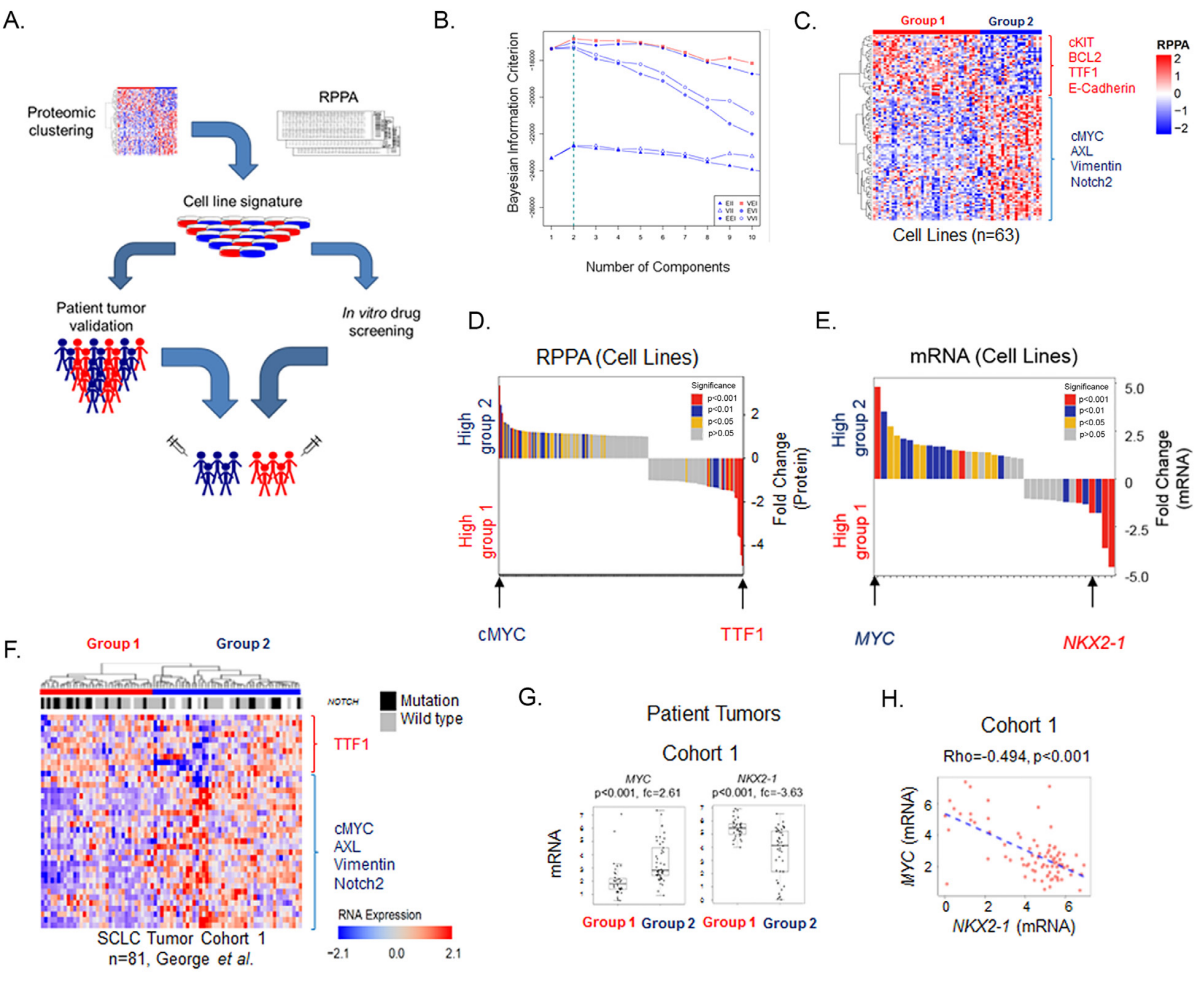
SCLC cell lines cluster into two subsets defined by TTF1 and cMYC **A.** Schematic flow of how SCLC was divided into two molecular subgroups using proteomic profiling of 63 cell lines and was subsequently utilized to identify molecular markers and potential therapeutic targets using clinical cohorts and drug sensitivity databases. **B.** Clustering using Bayesian Information Criterion (BIC) determines that two was the most significant number of clusters. **C.** Supervised hierarchical analysis shows distinct expression patterns between the two subsets which reflect the distinct protein expression patterns between them. **D.** Mean expression for each RPPA probe compared between the two subsets shows TTF1 and cMYC to be the most differentially expressed. **E.** Gene expression of significant total protein differences between the two subsets using publically available data available from 53 cell lines [29] shows *NKX2-1* and *MYC* to be amongst the most differentially expressed. **F.** Supervised hierarchical analysis of the expression of the 38 genes used in panel E in the George *et al.* patient cohort. Mutations in the *NOTCH* family (*NOTCH1, NOTCH2, NOTCH3* or *NOTCH4*) are also indicated. At the highest level patient samples fall into 2 groups based on cutting the first branch in the dendrogram as indicated by the blue and red bars. **G.** Comparison of cMYC/*MYC* and TTF1/*NKX2-1* expression between the two subsets in cell lines and patient samples. p-values determined by t-test.

To test the hypothesis that SCLC is a heterogeneous disease with distinct subtypes that can be defined at the proteomic level, we quantified the expression of 169 total- and phosphorylated-proteins (assayed by RPPA) in 63 SCLC cell lines. The optimal number of proteomic subgroups was then determined using a model-based clustering method [18, 19]. Specifically, six distinct models were used to classify cell lines into subgroups (between 1-20 subgroups defined by protein expression). Following this, Bayesian Index Criterion (BIC) was applied to determine the optimal number of subgroups. For all six models, maximum BIC scores were obtained when cell lines were divided into two groups (Groups 1 and 2) (Figure 1A-1B; Supplementary Figure 1). The optimal model/group combination was then used to segregate the cell lines into two groups. Global differences in protein expression between these two groups are illustrated in Figure 1C.

Having determined that at the highest level, each SCLC cell line could be assigned to one of two proteomic subsets, we then identified proteins driving subgroup membership. Comparing the mean expression of each protein between the two groups (Figure 1D), we found that the most highly expressed protein in Group 1 was TTF1 (FC = 3.4, *p* < 0.001), whereas the highest in Group 2 was cMYC (FC = 3.9, *p* < 0.001). Other proteins with differential expression include potentially druggable targets such as Bcl2 and cKIT (higher in Group 1: FC = 2.7 *p* < 0.001 and FC = 3.5, *p* < 0.001 respectively), and PARP and AXL (higher in Group 2: FC = 1.1 *p* = 0.024 and FC = 1.3, *p* < 0.001 respectively) (Figure 1D). Further supporting this two-group classification, an independent principal component analysis of the cell lines again identified two main clusters with cMYC and TTF1 as the most differentially expressed proteins (*p* < 0.001; Supplementary Figure 2).

To further validate these findings, we next tested whether protein differences between the Groups corresponded to differences in mRNA expression. For this analysis, we limited our analysis to the 38 total proteins differentially expressed between the Groups (*p* < 0.05, FC ≥1.3), as mRNA data would not reflect post-translational modifications detected by RPPA such as phosphorylation. Consistent with the protein findings, mRNA levels of *NKX2-1* (the gene name of TTF1) and *MYC* were among the most differentially expressed genes between the two cell line groups out of 38 tested (*NKX2-1* FC = -1.78 *p* < 0.001, *MYC* FC = 4.82 *p* < 0.001) (Figure 1E).

As cell culture may impact gene expression, we then tested whether the cell line findings were recapitulated in human SCLC tumors [20]. Because mRNA expression levels correlated well with total protein expression in the cell lines for most genes (e.g., correlation of TTF1 protein to *NKX2-1* mRNA levels Rho = 0.919, *p* < 0.001; cMYC protein and *MYC* mRNA Rho = 0.792, *p* < 0.001), we then used gene expression data from the same 38 genes used in Figure 1E to cluster patient tumors from two published cohorts (*n* = 81 and 23 respectively [16, 17]) (Figure 1F and Supplementary Figure 3). Based on differences in the expression of these 38 genes, at the highest level patient samples segregated into two groups (as indicated by the bars labelled groups 1 and 2), with the same group assignment for all 38 probes as seen with the cell lines (Figure 1E).

Similar to the cell lines, *NKX2-1* and *MYC* were among the most differentially expressed genes between the two groups (Figure 1G, Supplementary Figure 3). In Cohort 1 (George *et al),* we observed a 3.6-fold difference between groups for *NKX2-1* expression and a 2.6-fold difference between groups for *MYC*; in Cohort 2 (Sato *et al)* fold differences in *NKX2-1 and MYC* levels were 21.9 and 3.6, respectively, between the two main patient groups (all *p* < 0.001 by t-test). As expected, *NKX2-1* and *MYC* were also inversely correlated in both cell lines (Supplementary Figure 4) and patient tumors (Rho = -0.494, *p* < 0.001. Figure 1H). In contrast, there were no significant differences in commonly mutated genes (*p* < 0.05 by Fisher’s exact test). Mutations in the *NOTCH* family are shown in Figure 1F for illustrative purposes.

### TTF1-high SCLC is enriched for ASCL1

ASCL1 and NEUROD1 are largely non-overlapping transcription factors that are required for the normal development of multiple neuronal and neuroendocrine cell lineages [21] and have been used to define heterogeneity in SCLC [22]. ASCL1 has been associated with classic SCLC, whereas NEUROD1 has been associated with so-called variant SCLC [21, 23]. Prior studies have established that TTF1 and cMYC are targets of key SCLC transcription factors ASCL1 and NEUROD1 respectively [22]. Therefore, we tested whether expression of *ASCL1* and *NEUROD1* was significantly different between Groups 1 (TTF1 high) and 2 (cMYC high).

**Figure 2:**
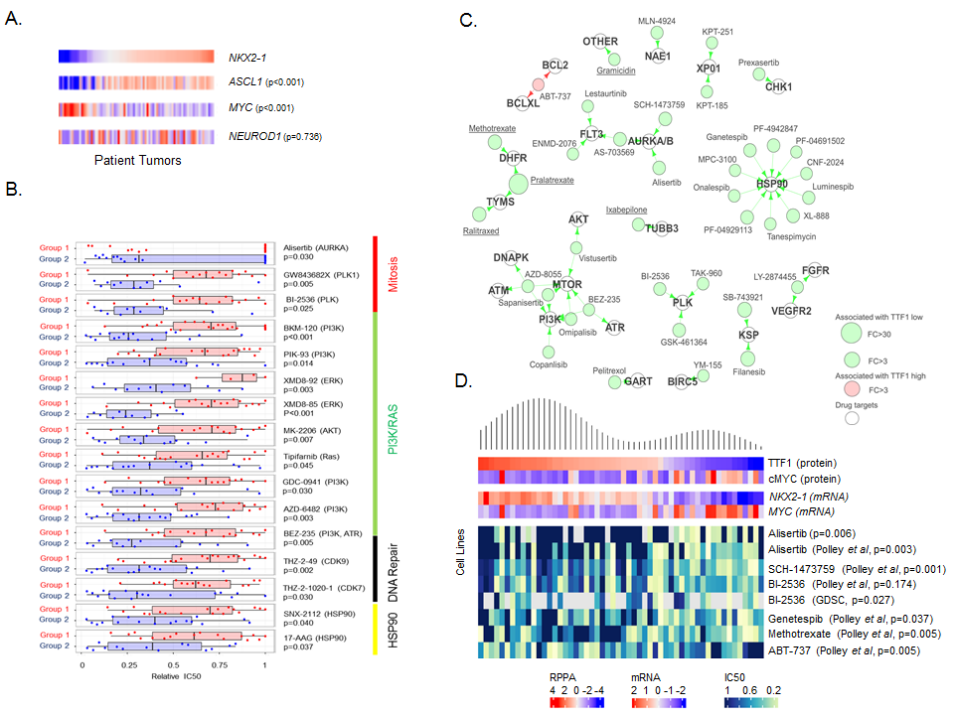
TTF1 and MYC are single biomarkers of response to multiple drugs **A.** Supervised comparison of *NKX2-1, ASCL1 MYC* and *NEUROD1* expression in George *et al.* patient tumors (sorted by *NKX2-1* expression, p-values for correlation to *NKX2-1*). **B.** Comparison of mean drug sensitivities (relative IC_50_) between the two subsets shows Group 2 (TTF1 low) cell lines to be more sensitive to a number of targeted agents. **C.** Drug-target constellation (DTECT) maps of drugs differentially sensitive between TTF1 high and low cell lines (FC >3.0, *p* < 0.01). Drugs with differential sensitivity are mapped by their primary, secondary and tertiary targets. Underlined drugs are either FDA approved or licensed for use in Canada/Europe. **D.** Density plot of TTF1 expression mapped to expression of cMYC, *NKX2-1, MYC* and sensitivity to selected drugs (relative IC_50_’s) in cell lines. p-values from Spearman correlation between TTF1 expression and IC_50_.

As expected, *ASCL1* gene expression was significantly higher in Group 1 (FC = -20.78, *p* < 0.001 cell lines, FC = -4.96, *p* < 0.001 patient tumors). However, *NEUROD1* expression was not significantly different between the Groups (cell lines *p* = 0.07, patient tumors *p* = 0.35). To further explore the role of ASCL1 and NEUROD1 and their relation to TTF1 and cMYC, we then ranked the SCLC lines and patient samples by *NKX2-1* expression and compared the expression of *ASCL1*, *MYC* and *NEUROD1* (Figure 2A). While a robust negative correlation was observed between *NKX2-1* and *MYC* (Rho = -0.570, *p* < 0.001 cell lines, Rho = -0.494, *p* < 0.001 patient tumors), as well as between *NKX2-1* and *ASCL1* (positively correlated, Rho = 0.601, *p* < 0.001 cell lines; Rho = 0.512, *p* < 0.001 patient tumors), no such correlation was seen with *NEUROD1* (Rho = 0.086, *p* = 0.465 cell lines; Rho = 0.178, *p* = 0.112 patient tumors).

We therefore conclude that, while ASCL1 overexpression likely explains mechanistically the high levels of TTF1 seen in Group 1, the proteomic subsets are not merely representative of the previously described classic and variant SCLC as they are not correlated with *NEUROD1* levels. Indeed, the lack of a negative correlation between *ASCL1* and *NEUROD1* (Rho = 0.004, *p* = 0.736 cell lines; Rho = 0.048, *p* = 0.668 patient samples)*,* along with findings from other groups that some SCLCs express neither *ASCL1* nor *NEUROD1* *[22]**,* would suggest the proteomic subsets provide additional information beyond the classic and variant designations. The cMYC high group is heterogeneous for NEUROD1 expression reflecting recent observations that there is an ASCL1/NEUROD1 double-negative subset of SCLC [23].

### TTF1 low SCLC is more sensitive to multiple classes of drugs

Having identified two subsets of SCLC, we then tested whether these subsets had different sensitivities to targeted therapies (many of which are in development for SCLC) or to standard chemotherapies. IC_50_ values to a total of 273 drugs were determined in our lab using proliferation assays in a panel of 51 cell lines or obtained from a public database (GDSC [24]). Differences in drug sensitivity between the Groups were determined by t-test. Group 2 (MYC high) was relatively more sensitive to 24 drugs (*p* < 0.05), including classes of drugs targeting mitosis (e.g., the AURKA inhibitor alisertib, polo-like kinase inhibitors GW843682X and BI-2536), PI3K/RAS (e.g., GDC-0941), HSP90 (e.g., 17-AAG), and DNA repair (e.g. BEZ-235 and THZ-2-49) (Figure 2B).

Having observed a differential sensitivity to drugs that target DNA repair such as BEZ-235, a PI3K/ATR inhibitor believed to kill cancer cells primarily through ATR inhibition [25] and the CDK inhibitors THZ-2-49 and THZ-2-1020-1 (CDK’s regulate the DNA damage response [26]), we compared our previously published DNA repair score between the two subsets [5]. The DNA repair score is modestly higher in Group 2 (Supplementary Figure 6) which may explain why Group 2 is more sensitive to DNA repair inhibition. Expression levels of the drug targets themselves were not significantly different between groups (all *p* >0.05), suggesting that for these agents the degree of target expression is not predictive of sensitivity. As platinum-based chemotherapy is standard of care treatment for SCLC, we also compared cisplatin sensitivity between the two subsets. Interestingly there was no significant difference in sensitivity to cisplatin (Supplementary Figure 6, *p* = 0.428) between the groups, or to other chemotherapeutic agents commonly used in the treatment of SCLC, including carboplatin (*p* = 0.149) etoposide (*p* = 0.405), irinotecan (*p* = 0.285), or topotecan (*p* = 0.938). Notably, none of the drugs analyzed had relatively greater activity in Group 1.

### TTF1 and cMYC single biomarker analysis

Within Group 2 SCLC patient tumors, we observed a subset with exceptionally high cMYC and low TTF1 (Figure 1F and Supplementary Figure 7). Based on this and the fact that protein expression of cMYC and TTF1 were the top markers defining proteomic subgroups in both cell lines and tumors, we then investigated whether TTF1 or cMYC, as individual markers, were predictive of *in vitro* drug response. Using a previously established method for determining bimodality [27], we found that the expression of these genes were bimodally distributed in both SCLC cell lines (bimodal index (BI) 2.34 and 1.86, for TTF1 and cMYC respectively) and patient tumors (1.85 and 1.55, respectively) (Supplementary Figure 7). Using the bimodal classification, we separated cell lines into TTF1-high (68%) and TTF1-low (32%) and cMYC-high (25%) and cMYC-low (75%).

Notably, cell lines with high cMYC expression were not exclusively *MYC* amplified. Indeed, only 7 of 13 cMYC over-expressing cell lines carry a known *MYC* amplification [4] (Supplementary Figure 8). High cMYC protein expression thus captures a larger subset of SCLC than *MYC* amplification alone. While *MYC* status is often reported in terms of *MYC* amplification [28], our findings suggest that cMYC expression levels (rather *MYC* amplification) identify a larger, translationally relevant subset of SCLC and is a more inclusive marker of MYC status.

We then compared drug sensitivity of TTF1-high versus TTF1-low cell lines using the recently published NCI SCLC drug sensitivity database [29]. This database contains sensitivity data for 63 human SCLC cell lines across 526 agents, including FDA-approved oncology agents and investigational agents. For this exploratory analysis, we identified those drugs with a greater than 3-fold difference in mean IC_50_ between TTF1-high and -low lines and a p-value ≤0.05 (equivalent FDR = 0.19) by t-test. Using this threshold, we identified 39 drugs, 38 of which were more effective in TTF1-low cell lines.

We subsequently applied an in-house curated drug-target database to generate a “drug-target constellation map” (DTECT map) of the 39 drugs with differential activity in cell lines with high versus low TTF1 levels (Figure 2C). Similar to the analysis of drug sensitivity between Groups 1 and 2, TTF1-low cell lines exhibited greater sensitivity to several drug classes, including drugs targeting aurora kinase (*n* = 3), PLK (*n* = 3), the PI3K/MTOR pathway (*n* = 6), and HSP90 (*n* = 10). For example, inhibitors of aurora kinase A/B with greater activity in TTF1-low cell lines included alisertib (FC = 7.3, *p* = 0.009), AS-703569 (FC = 8.1, *p* = 0.009), and SCH-1473759 (FC = 8.0, *p* = 0.001). A number of drugs included in the analysis are FDA approved for other cancer types (underlined in Figure 2C) and include the DHFR inhibitor methotrexate which has previously shown modest single agent activity in an unselected patient population with extensive stage SCLC [30].

Interestingly, the sole drug more effective in TTF1-high cell lines was the Bcl2 inhibitor ABT-737, which has been shown to have activity in SCLC PDX models in combination with rapamycin (mTOR inhibitor) [31]. The targets of ABT-737 (Bcl2 and BCLXL) are the only targets of drugs identified in this analysis to have significantly different expression between TTF1 high and low cell lines (FC = 2.85, *p* = 0.008, and FC = 2.13, *p* = 0.040 respectively, higher in the TTF1 high/ABT-737 sensitive group). An association between higher Bcl2 expression and sensitivity to Bcl2 inhibition has previously been described in both SCLC and lymphoma models [32, 33]. To further visualize the relationship between drug sensitivity and biomarker expression at the individual cell line level, we generated a heatmap in which cell lines were ordered by TTF1 protein expression and correlations with cMYC expression and sensitivity to top drugs from DTECT analysis are shown (including drugs targeting aurora kinase, PLK, and BCL-2) (Figure 2D).

We also analyzed drug sensitivity based on cMYC expression (Supplementary Figure 9). Applying the same criteria as for TTF1, we identified 11 drugs, all of which were more effective in cMYC high cell lines. Eight of the drugs identified inhibited the same targets identified in the TTF1 analysis (e.g. aurora kinase, PLK, DHFR). For example, the aurora kinase inhibitor AS-703569 was found to be more efficacious in cMYC-high cell lines (FC = 6.8, *p* = 0.034), as were the PLK inhibitors MLN-0905 (FC = 5.9, *p* = 0.019) and TAK-960 (FC = 3.0, *p* = 0.040). Interestingly, one of the three drugs unique to the cMYC analysis of the NCI database was the CHK1 inhibitor praxasertib (LY-2606368) (FC = 1.9, *p* = 0.006), which our group has independently been shown to have preferential activity in pre-clinical models of SCLC that overexpress cMYC protein [4].

### cMYC protein predicts response to the aurora kinase A inhibitor alisertib

The aurora kinase A inhibitor alisertib has demonstrated preclinical and/or clinical activity in several cancer types, including SCLC and other high grade neuroendocrine cancers such as neuroblastoma [34]. Based on promising activity of single agent alisertib in a subset of SCLC patients [35], the combination of alisertib with paclitaxel was recently investigated in a Phase 2 clinical trial for relapsed SCLC (NCT02038647). However, there are no established predictive biomarkers for this drug.

**Figure 3:**
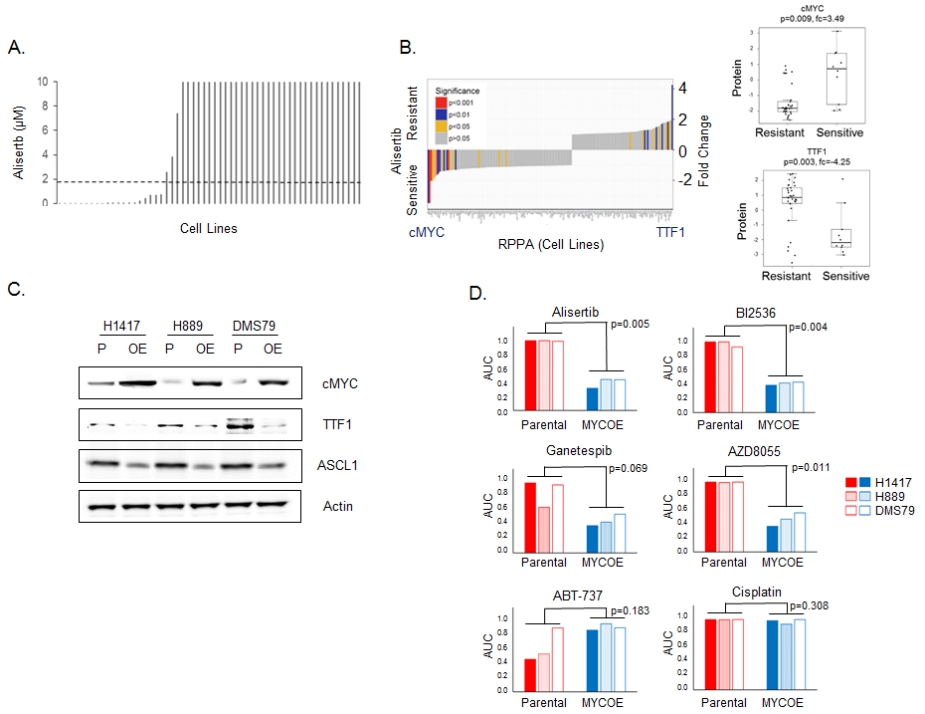
TTF1 and cMYC are biomarkers of response to alisertib *in vitro* **A.** Cell line sensitivity to alisertib (ranked by IC_50_, µM), dashed line indicates Cmax (1.83µM) from Phase 1 study [47]. **B.** Comparison of proteomic markers between most (IC_50_ < 0.1µM) and least sensitive (IC_50_≥10µM) cell lines. p-values by t-test. **C.** Western blot analysis comparing protein expression between parental (P) and cMYC overexpressing (OE) cell lines. **D.** Comparison of drug sensitivity between parental and cMYC overexpressing (MYCOE) cell lines. Area under the curve (AUC) calculated from dose response curves and compared by paired test between the parental and MYCOE groups.

We performed a supervised analysis of candidate proteomic biomarkers for single agent alisertib in our cell line panel using the full RPPA panel. 51 SCLC cell lines were treated with alisertib for four days and IC_50_ values calculated (Figure 3A, range 0.002µM to >10µM, median = 10µM, mean = 6.27µM). We then compared expression of protein markers between the most (IC_50_ < 0.1µM, n = 15) and least sensitive (IC_50_ ≥10 µM, *n* = 33) cell lines (Figure 3B) and as continuous variables.

Comparing the most sensitive versus the least sensitive cell lines, cMYC (FC = 3.49, *p* = 0.009) and TTF1 (FC = -4.25, *p* = 0.003) were identified as the top markers of resistance and sensitivity, respectively. Similarly, when compared as continuous variables, both cMYC and TTF1 were highly correlated with alisertib sensitivity (cMYC: Rho = -0.450, *p* < 0.001; TTF1: Rho = 0.385, *p* = 0.006). While the association of *MYC* amplification with sensitivity to alisertib is not new observation [23, 28], we identify twice as many SCLC cell lines that are sensitive to alisertib based on high cMYC expression (14 cell lines) than would be identified by *MYC* amplification alone (7 cell lines). This finding - that high cMYC protein expression and low TTF1 protein expression are top markers of alisertib sensitivity (out of 170 protein markers tested) through a supervised analysis of alisertib is consistent with the identification of aurora kinase inhibitor activity in the DTECT analyses above.

The recent publication by Mollaoglu *et al* confirmed MYC as a driver of response to alisertib by showing that over-expression of MYC in a conditional genetically engineered mouse model (GEMM) of SCLC with *Myc* overexpression increased sensitivity to alisertib [23]. To further understand the functional role cMYC plays in the sensitivity of SCLC to targeted therapeutics, we created stable clones of three human SCLC cell lines (H889, H1417 and DMS79) that overexpress cMYC. Western blot analysis comparing parental and cMYC overexpressing cell lines showed in all cases an increase in cMYC expression and decreases in TTF1 and ASCL1 expression (Figure 3C).

We hypothesized that altering cMYC expression would affect sensitivity to a panel of targeted agents identified in our earlier analyses - increasing sensitivity to alisertib, BI2536, ganetespib and AZD8055; reducing sensitivity to ABT-737; but having no effect on cisplatin sensitivity. As predicted, cMYC overexpressing clones showed greater sensitivity to alisertib (*p* = 0.005), BI2536 (p = 0.004), ganetespib (*p* = 0.069) and AZD8055 (*p* = 0.011) (Figure 3D). In contrast but as predicted, in the two parental cell lines that were sensitive to the Bcl2 inhibitor ABT-737 (H1417 and H889), cMYC overexpression lead to decrease in sensitivity (although this did not reach statistical significance (*p* = 0.183) as the DMS79 parental cell line was not sensitive). Notably, altering cMYC expression had no effect upon sensitivity to cisplatin, confirming our earlier observation in the proteomic subset analysis (Supplementary Figure 6) and indicating that the differences in drug sensitivity are not merely due to differences in the rate of cell proliferation driven by elevated cMYC. These experiments suggest that cMYC expression has a direct effect on both cellular signaling and drug sensitivity.

### TTF1 as a surrogate marker for DLL3 expression

The antibody-drug conjugate (ADC) rovalpituzumab tesirine that delivers a pyrrolobenzodiazepine dimer toxin to DLL3 expressing cells has shown striking clinical activity in relapsed, DLL3-expressing SCLC (NCT0191653) [36]. Based on this, a registration study in DLL3-positive, relapsed SCLC is currently underway (TRINITY trial, NCT02674568). DLL3, an inhibitory NOTCH ligand, is overexpressed in many neuroendocrine cancers and is a downstream target of ASCL1 [37]. As NOTCH2 protein expression was observed to be higher in group 2 (TTF1 low), while ASCL1 was higher in group 1 (TTF1 high), we investigated whether the proteomically defined subsets would also delineate SCLC subsets with different expression of DLL3 and, therefore, vulnerability to rovalpituzumab tesirine.

**Figure 4:**
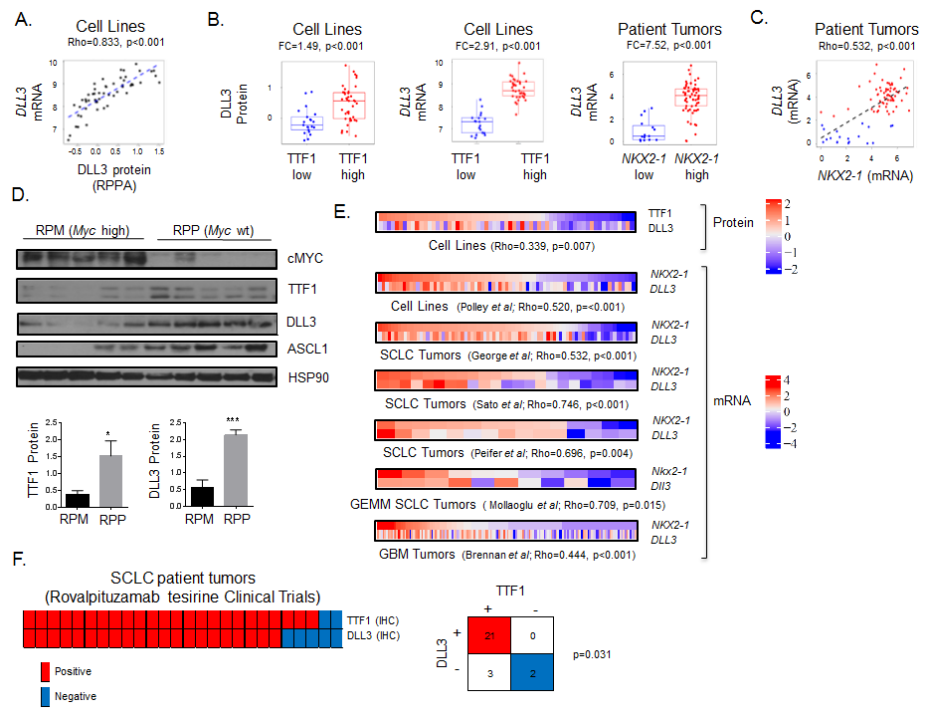
TTF1 is a surrogate marker of DLL3 expression **A.** Spearman correlation of DLL3 protein and *DLL3* gene expression in SCLC cell lines. **B.** Comparison of DLL3 protein and *DLL3* gene expression between TTF1 high/low cell lines (bimodal), and *DLL3* gene expression between *NKX2-1* high/low patient samples. p-values by t-test. **C.** Correlation between *NKX2-1* and *DLL3* gene expression in patients from the George *et al* cohort color coded for high (red) and low (blue) DLL3 based on bimodal distribution (BI = 0.92). Correlation of TTF1 H-score with DLL3 protein and *DLL3* gene expression in SCLC PDX models (color coded for TTF1 positive (blue, IHC score≥1) and negative (IHC score < 1). **D.** Western blot analysis comparing cMYC, TTF1, DLL3 and ASCL2 expression between SCLC GEMM models with and without cMYC overexpression (RPM and RPP respectively). TTF1 and DLL3 expression relative to HSP90 quantified (p-values by t-test, * *p* < 0.05, *** *p* < 0.001). **E.** Heatmaps comparing TTF1 and DLL3 protein expression in SCLC cell lines and comparing *NKX2-1* and *DLL3* gene expression in SCLC cell lines, three SCLC clinical cohorts (George *et al.,* Sato *et al.,* and Peifer *et al.*) and one glioblastoma clinical cohort (Brennan *et al.*). p-values from Spearman correlation between TTF1 and DLL3 or *NKX2-1* and *DLL3*. Gene expression data standardized to same scale across datasets. **F.** Comparison of TTF1 and DLL3 IHC staining in 26 patients screened for enrollment in trials at MD Anderson Cancer Center. Fisher’s exact test shows significant concordance between staining for the two markers.

Published data from our group has demonstrated a strong positive correlation between levels of TTF1 protein as measured by IHC and RPPA and between TTF1 protein levels and *NKX2-1* mRNA in lung tumors [38]. Similarly, here we found that DLL3 protein (by RPPA) and *DLL3* mRNA levels were highly correlated in SCLC cell lines (Rho = 0.833, *p* < 0.001, Figure 4A). Categorizing cell lines as TTF1-high versus TTF1-low based on bimodal protein expression revealed higher DLL3 protein and mRNA expression in the TTF1 high group (Figure 4B. DLL3 protein: FC = 1.49; *DLL3* gene expression: FC = 2.91; both *p* < 0.001). Similarly patient samples with high *NKX2-1* mRNA expression also had higher *DLL3* expression (Figure 4B, FC = 7.52, *p* < 0.001), whereas *NOTCH* family members (*NOTCH1, NOTCH2, NOTCH3*) were higher in *NKX2-1*-low tumors (FC ≥ 1.28, *p* ≤ 0.005).

Based on these results, we tested the correlation between *NKX2-1* and *DLL3* mRNA expression in patient samples, finding that *NKX2-1* and *DLL3* were strongly correlated (Rho = 0.532, *p* < 0.001. Figure 4C). In a panel of SCLC PDX models [39] we correlated TTF1 and DLL3 protein expression (R = 0.499, *p* = 0.083). Although this was not statistically significant likely due to the small sample size, it does reinforce the association between TTF1 and DLL3.

We then tested whether these observations from the human models/patients were recapitulated in the genetic SCLC (GEMM) models. We hypothesized that expression of TTF1, DLL3 and ASCL1 would be higher in the *Myc* wild type (RPP) GEMM model than in the *Myc* overexpressing model (RPM). Western blot analysis comparing five RPM and five RPP tumors showed significantly higher expression of TTF1, ASCL1, and DLL3 in the *Myc* wild type models (Figure 4D, TTF1 *p* = 0.041, DLL3 *p* < 0.001).

We further compared the correlation between TTF1 and DLL3 protein in cell lines, and *NKX2-1* and *DLL3* mRNA in cell lines, an additional SCLC patient cohort [40] and a cohort of glioblastoma patient samples [41] and found significant correlations in each dataset (Figure 4E). The correlation between *DLL3* and *NKX2-1* in glioblastoma is notable as rovalpituzumab tesirine is indicated for glioblastoma in an ongoing basket trial (NCT 02709889) and requires DLL3 expression for enrollment. We also compared expression of *Nkx2-1* and *Dll3* in the *Myc* overexpressing (RPM) GEMM model of SCLC [23], where we observed a strong correlation (Rho = 0.709, *p* = 0.015). The overexpression of *Myc* in this model generated a heterogeneous population of tumors allowing for comparison of the relationship between *Nkx2-1* and *Dll3* in a consistent genetic back ground.

To validate the potential for TTF1 IHC to substitute for DLL3 IHC we compared staining of these markers in patients screened for enrollment in the recently published phase I [36] and the TRINITY trial testing rovalpituzumab tesirine in SCLC (Figure 4F). For the six SCLC patients treated on the phase I study and the 25 treated on the TRINITY trial at MD Anderson, IHC scores (positive or negative) were available for both markers in a total of 26 patients. Concordant staining, either positive or negative for both TTF1 (from diagnostic pathology reports) and DLL3 was observed in 23 of the 26 patients (*p* = 0.031 by Fishers exact test, Figure 4F). Combined, these findings suggest that TTF1 protein expression (a routine clinical diagnostic IHC marker for lung cancer) could be a surrogate marker for DLL3 expression and, thus, help identify patients with DLL3-positive tumors (which have the highest response rates to rovalpituzumab tesirine).

## DISCUSSION

Subtyping of cancers has shown potential to both increase our understanding of cancer biology and to identify sub-populations with greater response to particular therapeutic agents [9, 38, 42]. In this study we investigated proteomic subtyping of SCLC using cell lines and patient samples. Using a unique dataset of proteomic profiles across 63 cell lines, we initially found that SCLC can be separated into two main subsets defined by levels of TTF1 and cMYC expression. Using publically available data from two clinical cohorts we validated these groups in patient samples. Major findings in this study include an association between protein expression of TTF1 and/or cMYC expression and (1) sensitivity to alisertib and other mitotic inhibitors, and (2) a strong, positive correlation between DLL3 expression (the target of and marker of response to rovalpituzumab tesirine) and TTF1.

In-house and publically available SCLC drug screening data allowed us to investigate those targeted agents with different activity between proteomic subsets. We found that cell lines in Group 2 had greater sensitivity to multiple agents including those inhibiting aurora kinase (e.g., alisertib), PLK, PI3K/RAS pathway, and DNA repair. This analysis was further refined using single markers - TTF1 and cMYC protein - and linking these to *in vitro* sensitivity. This analysis highlighted the ability for these single markers to predict sensitivity or resistance to several key drug classes currently in development for SCLC. These include Bcl2 inhibitors, mitotic inhibitors (aurora kinase and PLK), and CHK1 inhibitors.

Interestingly, the observation that TTF1 low expressing SCLC are more sensitive to inhibitors of DNA repair mirrors our previous findings in lung adenocarcinoma [38], suggesting a common effect of TTF1 loss in SCLC and LUAD in regards to DNA damage response. This finding may be related to higher cMYC levels (and therefore greater replication stress), as higher cMYC levels were observed in LUAD and SCLC with low TTF1. While single marker analysis using TTF1 recapitulated many of the observations from the initial clusters (Groups 1 and 2), it also identified additional vulnerabilities including greater activity of HSP90 inhibitors in TTF1-low SCLC and of Bcl2 inhibitors in TTF1-high models. These findings suggest that a patient selection strategy based on TTF1 status might enrich for patients more likely to respond to Bcl2 inhibition, despite previous negative trial results in an unselected SCLC patient population [8].

The recent phase 1 study of rovalpituzumab tesirine, an antibody-drug conjugate targeting DLL3, found that SCLC patients whose tumors express DLL3 had higher response rates [36]. Our analysis of human cell lines, multiple patient cohorts (including both SCLC and glioblastoma), and mouse models revealed a strong, positive correlation between the expression of DLL3 and TTF1. Although the SCLC samples analyzed here are predominantly from early stage surgical resections, we observe a similar percentage of TTF1-low tumors (18.5%) as reported elsewhere in advanced disease (17.2%)[43]. Clinically, concordance between TTF1 staining (from diagnostic pathology reports) and DLL3 IHC scores in tumors from patients treated with rovalpituzumab tesirine at our institution further confirmed this observation. TTF1 IHC is a diagnostic biomarker used routinely in the clinic to discriminate primary lung tumors versus metastases from other sites and to determine histologic subtype (e.g., SCLC, adenocarcinoma, squamous). Based on the findings here, TTF1 IHC may have a clinical application as a biomarker to identify those patients who should be screened for DLL3 expression for enrollment onto rovalpituzumab tesirine trials or for other DLL3 targeted therapies.

Using two approaches, we show that cMYC expression is the top marker of response to alisertib. This observation agrees with a recently published study in which a GEMM model of SCLC that overexpresses cMYC was more sensitive to alisertib than the cMYC wild-type model [23]. Initial biomarker analysis from the recent clinical trial comparing chemotherapy (paclitaxel) alone versus chemotherapy with alisertib (which did not meet its primary endpoint of improved progression free survival (PFS) in unselected SCLC patients) has been completed [44]. Based on the preclinical findings described here, an initial analysis of cMYC expression by IHC was performed that indicated significantly higher PFS in cMYC positive patients receiving alisertib (*p* < 0.001). Further analysis of patients and related biomarkers from these studies are ongoing. However, if confirmed, these findings would provide clinical validation of the pre-clinical observations described here.

**Figure 5:**
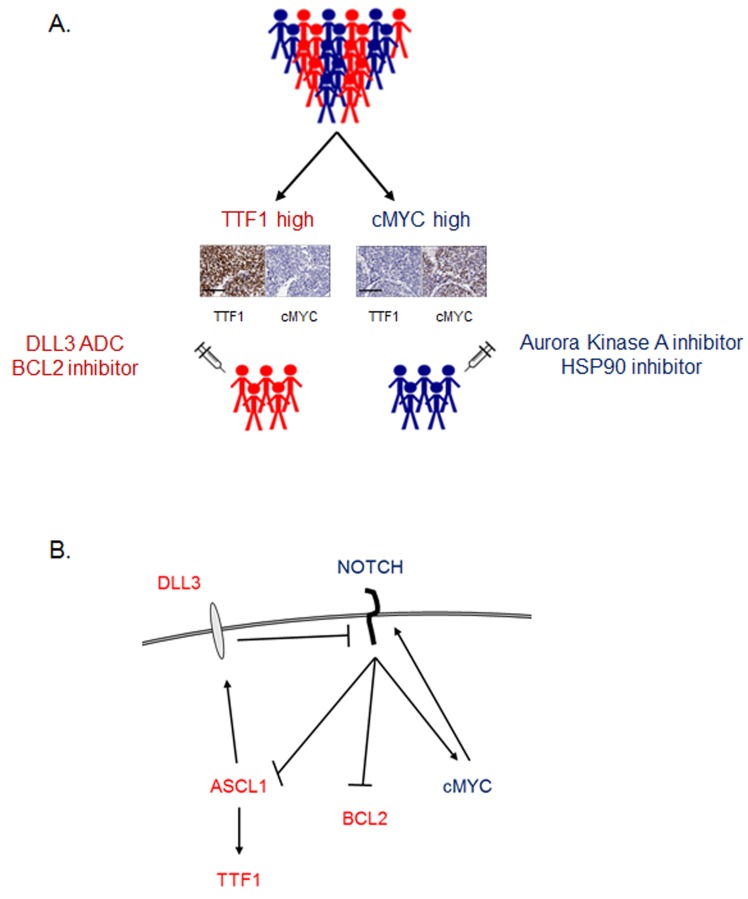
Working model **A.** Working model of how SCLC patients may be divided into two groups based on TTF1 and cMYC IHC, with different therapeutic vulnerabilities between the two groups. Example TTF1 and cMYC IHC from two archived SCLC tumor samples on a neuroendocrine TMA (scale bar = 100µm) **B.** Schematic of signaling pathways integrating how key molecules identified in these studies may interact to result in TTF1 high and cMYC high subsets. Proteins/genes in red have higher expression in TTF1 high SCLC, those in blue in cMYC high SCLC.

This study identifies a subset of SCLC characterized by high TTF1 expression that has an overall protein expression pattern distinct from TTF1-low SCLC. TTF1 is under the control of a second transcription factor ASCL1 [21] and while ASCL1 is elevated in the TTF1-high subset, we observe significant heterogeneity at the protein level in ASCL1 negative SCLC. This heterogeneity in the ASCL1 negative group demonstrates that the differences observed at the mRNA level are associated with a distinct profile going beyond the previously reported classic (ASCL1) versus variant (NEUROD1) classification of SCLC [22, 23]. Expression of ASCL1 is inhibited by NOTCH family signaling [45], which is down-regulated in the TTF1 subset, possibly through the over-expression of DLL3 (which itself is a downstream target of ASCL1 [37]) providing a potential mechanism through which TTF1 is overexpressed [46]. When cMYC (both a downstream target of NOTCH and a promoter of NOTCH signaling) is overexpressed in either cell lines or the GEMM model, expression of ASCL1 and TTF1 is significantly reduced indicating that cMYC overexpression drives membership of the cMYC high subset. A working model of how these molecules may interact is shown as Figure 5.

In summary, the subsets shown here can be identified clinically using an established, standard-of-care for lung cancer, biomarker (TTF1) or a marker routinely used in other cancers such as breast cancer (cMYC). These markers - particularly TTF1 - could be easily translated into clinical practice and present novel candidate predictive markers that could be immediately leveraged for chemo-refractory SCLC patients to select treatment with rovalpituzumab tesirine (TTF1-positive) versus other targeted agents such as alisertib (TTF1-negative, cMYC-high).

## MATERIALS AND METHODS

### Clinical cohorts

The George *et al.* cohort [16] included material from 152 SCLC cases (predominantly treatment naïve stage I-IV tumors) obtained by surgical resection (132), plural effusion (1) or autopsy (15). Gene expression data was available for 81 patients.

The Sato *et al.* cohort [17] included material from 23 SCLC cases obtained from patients undergoing pulmonary resection.

The Peifer *et al* cohort [40] included material from 15 SCLC cases obtained from patients undergoing surgical resection.

The Brennan *et al* cohort [41] included material from 164 glioblastoma cases.

### Patient population

Patients enrolled in the rovalpituzumab tesirine clinical trials provided written consent for participation and the study was approved by The MD Anderson Cancer Center Institutional Review Board.

### Biostatistics

BIC criteria: We estimated the number of clusters using model-based clustering method based on Gaussian mixture models [18, 19]. It uses maximum likelihood to fit models with different covariance matrix parameterizations over a range of different components. Then the best model is selected based on Bayesian Information Criterion (BIC). Maximum value of BIC score indicates optimal model with corresponding number of clusters and covariance matrix structure.

Bimodal Index: The bimodality index [27] was used to identify genes and proteins with bimodal expression patterns.

Additional methods are included in the supplemental materials.

## SUPPLEMENTARY MATERIALS FIGURES


