# Supplementary material for: Protein expression of TTF1 and cMYC define distinct molecular subgroups of small cell lung cancer with unique vulnerabilities to aurora kinase inhibition, DLL3 targeting, and other targeted therapies
Source: Oncotarget. 2017 Sep 1;8(43):73419–32. doi: 10.18632/oncotarget.20621 (PMC5650272; doi:10.18632/oncotarget.20621)
Supplement: Supplementary file 1 [file oncotarget-08-73419-s001.pdf]

Protein expression of TTF1 and cMYC define distinct molecular subgroups of small cell lung cancer with unique vulnerabilities to aurora kinase inhibition, DLL3 targeting, and other targeted therapies

SUPPLEMENTARY MATERIALS

Cell lines

Human SCLC cell lines COR-L88, DMS 114, DMS 153, DMS 273, DMS 53, DMS 79, H1048, H1092, H1105, H1184, H1238, H128, H1341, H1417, H1436, H146, H1522, H1618, H1672, H1688, H1694, H1836, H187, H1876, H1930, H196, H1963, H2081, H209, H211, H2107, H2108, H2141, H2171, H2195, H2196, H2198, H2029, H220, H2227, H2330, H250, H345, H378, H446, H510, H524, H526, H660, H69, H69/CPR, H719, H735, H740, H748, H774, H82, H841, H847, H865, H889, and SHP-77 were obtained from ATCC (Manassas, VA) or Sigma-Aldrich (St. Louis, MO); and human patient-derived xenograft (PDX) derived cell line NJH29 generously provided by Dr. Julien Sage (Stanford University, Stanford CA). All cells were grown in suggested medium supplemented with FBS and penicillin/streptomycin. Cells were passaged for fewer than 6 months following receipt. Cell line authenticity was validated by STR testing.

Reverse phase protein array

Protein lysates were collected and the slide images were quantified as previously described (Byers et al., 2013; Byers et al., 2012). The raw concentration data were normalized by median-centering each sample across all the proteins to correct loading bias.

Proliferation assays

Cells were seeded in 96-well plates at 2,000 cells per well. After 24 hours, the cells in each well were treated with alisertib, olaparib, BKM-120, amuvatinib, EPZ005687, ABT-737, AZD8055, ganetespib, BI2536, (Selleck Chemicals, Houston TX), talazoparib (BioMarin Pharmaceuticals), LY2606368, cisplatin or with vehicle control. Four days later proliferation was assayed by Cell Titer Glo (Promega, Fitchburg, WI). In-house drug response curves, IC_{50} values and AUC values were generated using the drexplorer package (Tong et al., 2015). Drug resistance data (IC_{50} value) was also downloaded from the GDSC website (Yang et al., 2013) and from the NCI SCLC drug sensitivity website (Polley et al., 2016).

Cell Line cMYC overexpression

H889, DMS-79 and H1417 were transformed with Dharmacon Precision LentiORF MYC viral particles (CloneID: PLOHS_1000008545). Transformed clones were selection by puromycin selection. Overexpression was confirmed by western blot.

Western blot

For cell lysates: Nitrocellulose western blots were probed for actin (sc1616), ASCL1 (sc390794) (Santa Cruz Biotechnology), Dallas TX), cMYC (cs9402), (Cell Signaling Technology, Danvers MA) and TTF1 (5883-1) (Epitomics). IR-conjugated anti-rabbit and anti-goat antibodies were used for detection.

For primary lung tumor lysates from the RPM (*Rb1/p53/Myc*) and RPP (*Rb1/p53/Pten*) models PVDF membranes were probed for. MYC (Cell Signaling #13987, 1:1000), NEUROD1 (Cell Signaling #4373), ASCL1 (BD Pharmingen #556604), DLL3 (Cell Signaling #2483), TTF1 (Abcam #ab76013), HSP90 (Cell Signaling #4877). For detection, membranes were incubated with Mouse and rabbit HRP-conjugated secondary antibodies (Jackson ImmunoResearch) and exposed to WesternBright HRP Quantum substrate (Advansta) .

Relative quantification of bands from immunoblot films were calculated using ImageJ. Values represent the pixel density for the band of each target protein relative to the sample loading control (HSP90). Statistical analysis was performed using GraphPad Prism with Student's unpaired t test,

Mouse RNA-Seq data

Mouse lung tumor RNA-seq data including 11 RPM tumors were obtained from Mollaoglu et al, 2017 (GEO: GSE89660). Sample S105-5 was excluded from analysis due to low RIN value. Mouse mm10 annotations (Ensembl build 82) were used in the RSEM (v1.2.12) utility rsem-prepare-reference to create bowtie (v1.0.1) indices. Gene expression was determined using the RSEM utility rsem-calculate-expression with the forward strand probability set to zero. Differential expression was determined using EBSeq (v1.4.0) using 'MedianNorm' function to calculate size factors and setting 'maxround' to 10.

Immunohistochemistry

Immunohistochemical staining for DLL3 was performed as reported previously (Rudin et al, 2017).

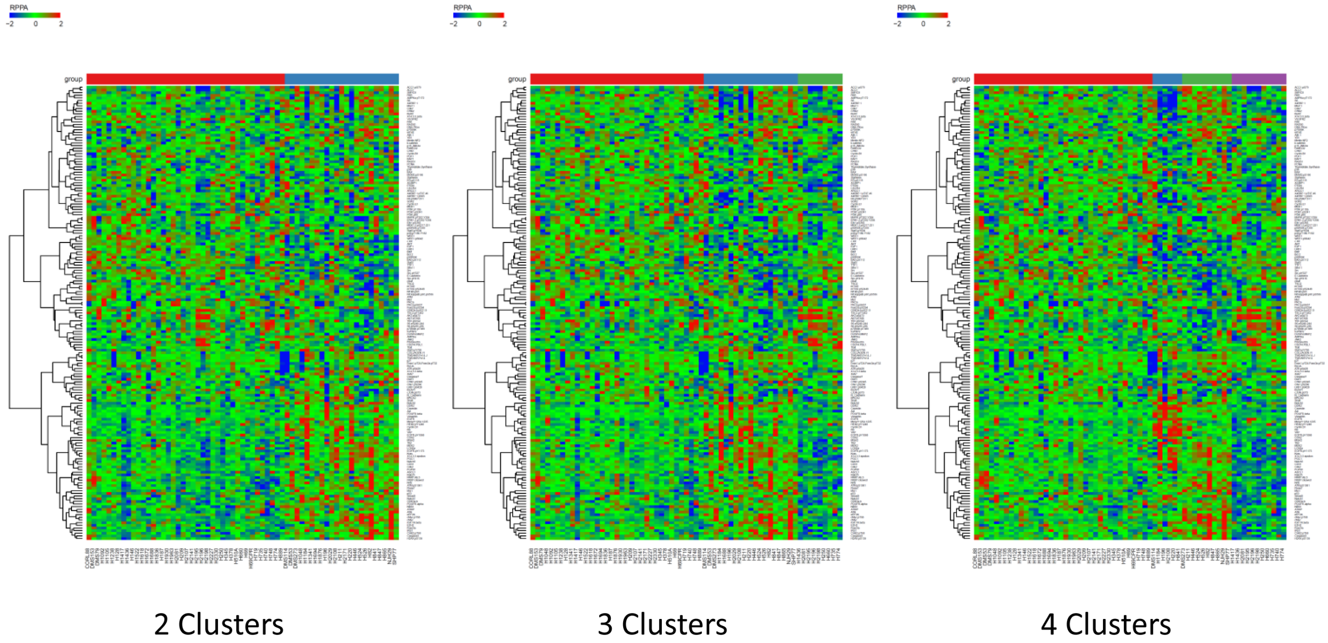

Supplementary Figure 1: Supervised hierarchical analysis of protein expression patterns with up to four clusters.

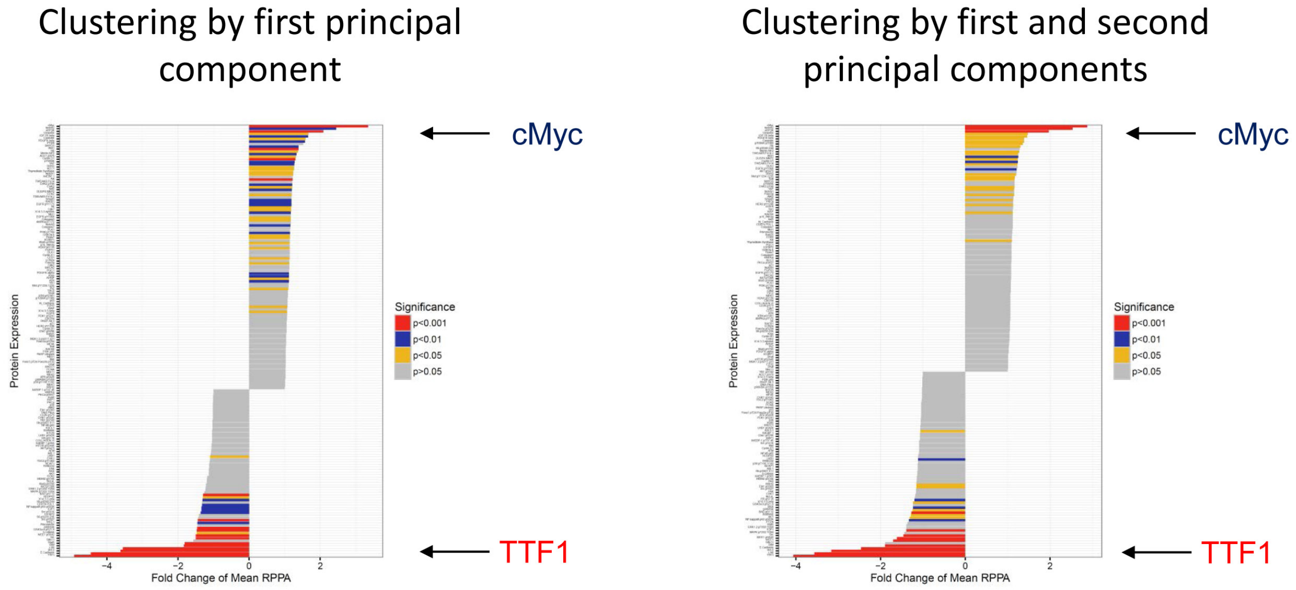

Supplementary Figure 2: Comparison of protein expression between subsets of SCLC cell lines clustered using the first principal component, or the first and second principal components identifies TTF1 and cMYC as the most differentially expressed proteins.

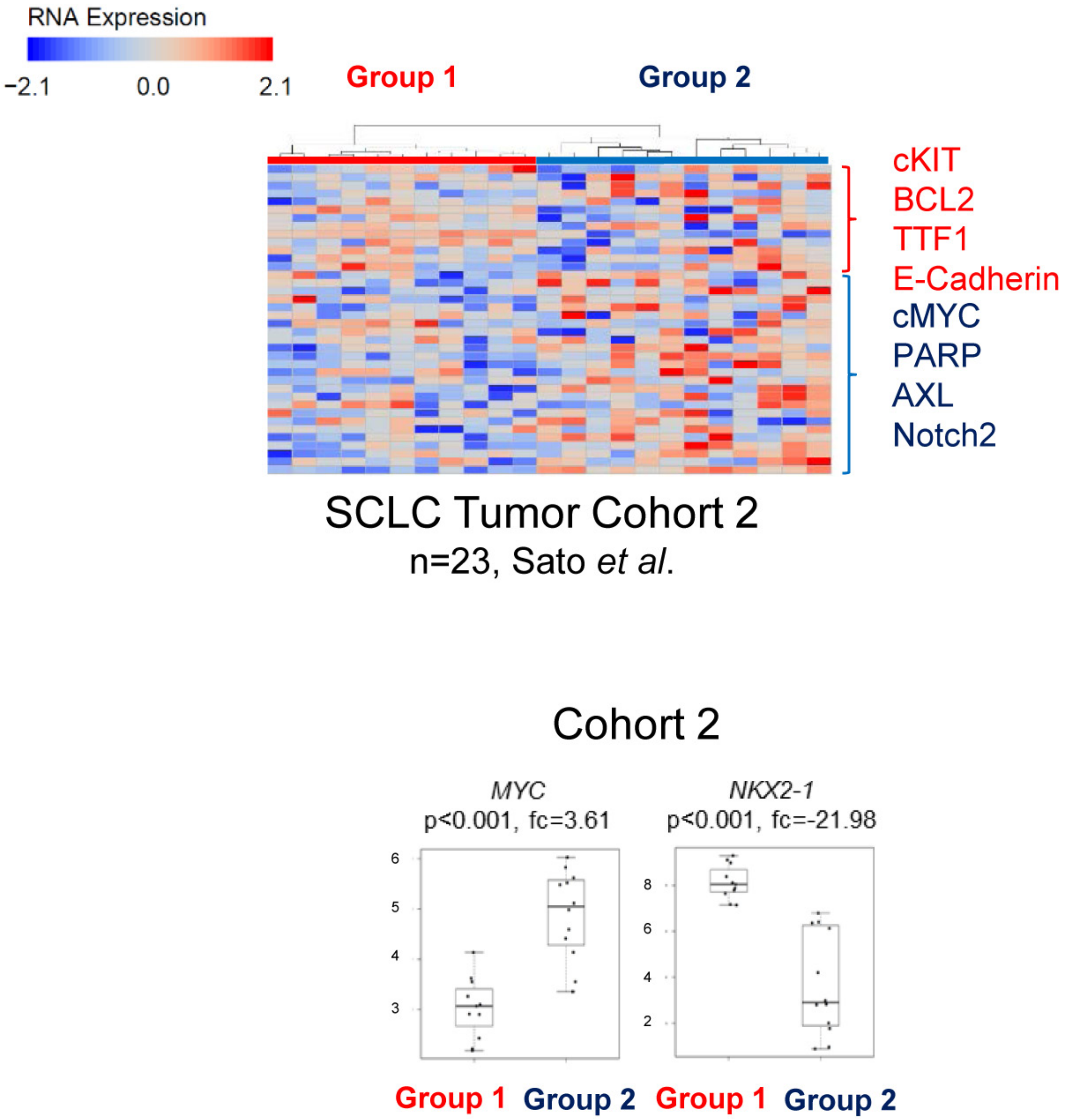

Supplementary Figure 3: TTF1 and cMYC are negatively correlated in SCLC cell lines. A. Spearman correlation between TTF1 and cMYC protein levels by RPPA. **B.** Spearman correlation between NKX2-1 and MYC gene expression.

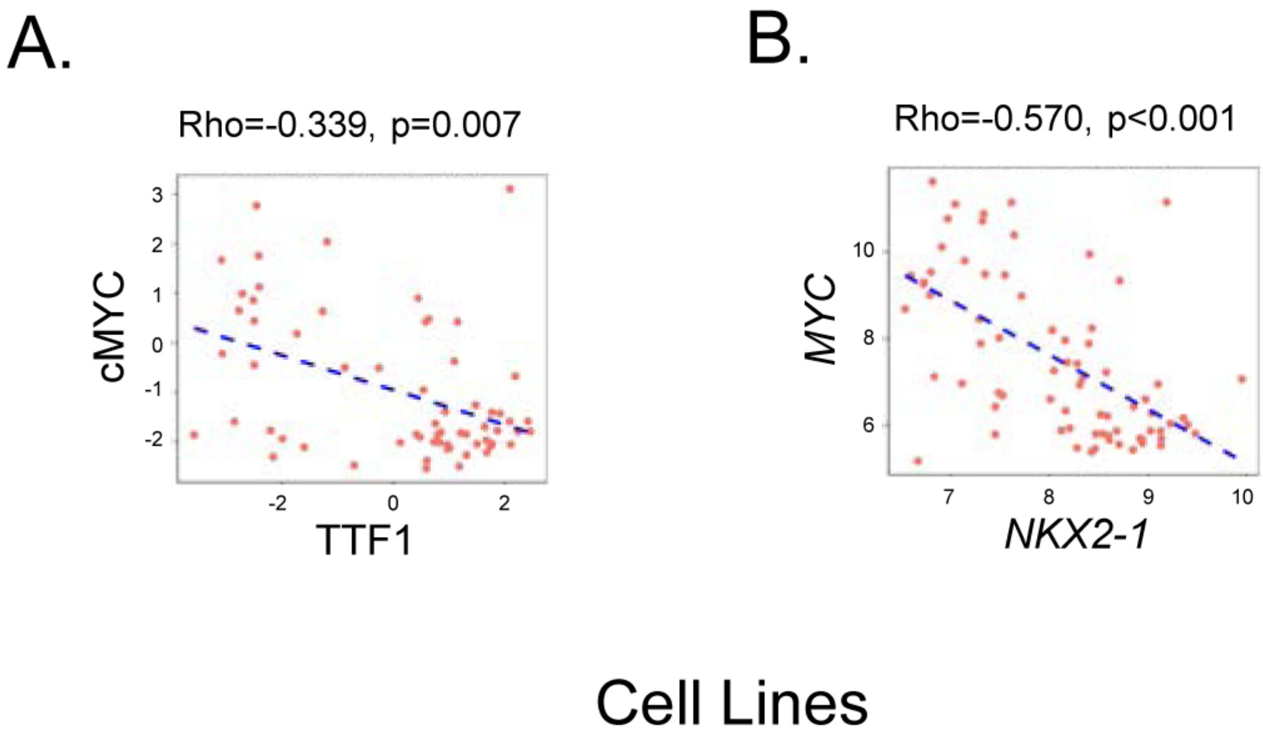

Supplementary Figure 4: Supervised comparison of *NKX2-1, ASCL1, MYC* and *NEUROD1* expression in SCLC cell lines.

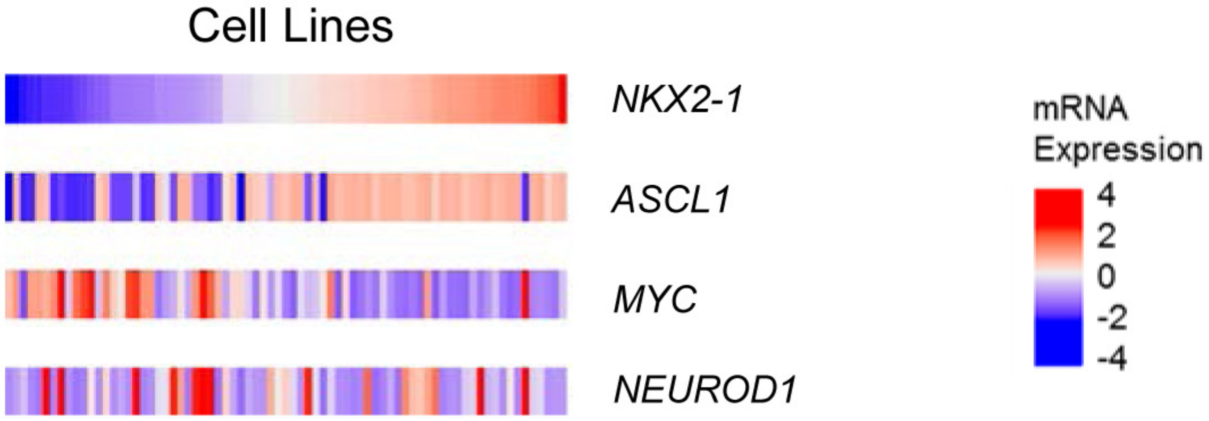

Supplementary Figure 5: Expression of TTF1 and cMYC is bimodal in cell lines and patient tumors. A. The DNA repair score (Cardnell et al., 2013) is higher in Group 2. p-values determined by t-test. **B.** The two groups are similarly sensitive to cisplatin (C). p-values determined by t-test.

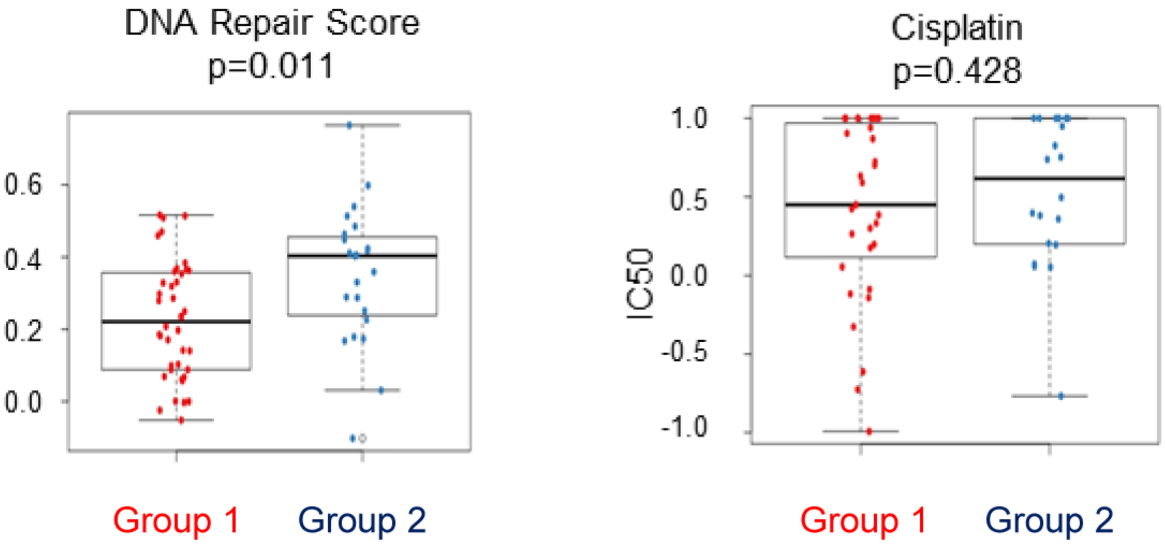

Supplementary Figure 6: cMYC protein and MYC gene expression are highly correlated in SCLC cell lines. Spearman correlation between cMYC protein and MYC gene expression color coded by MYC amplification.

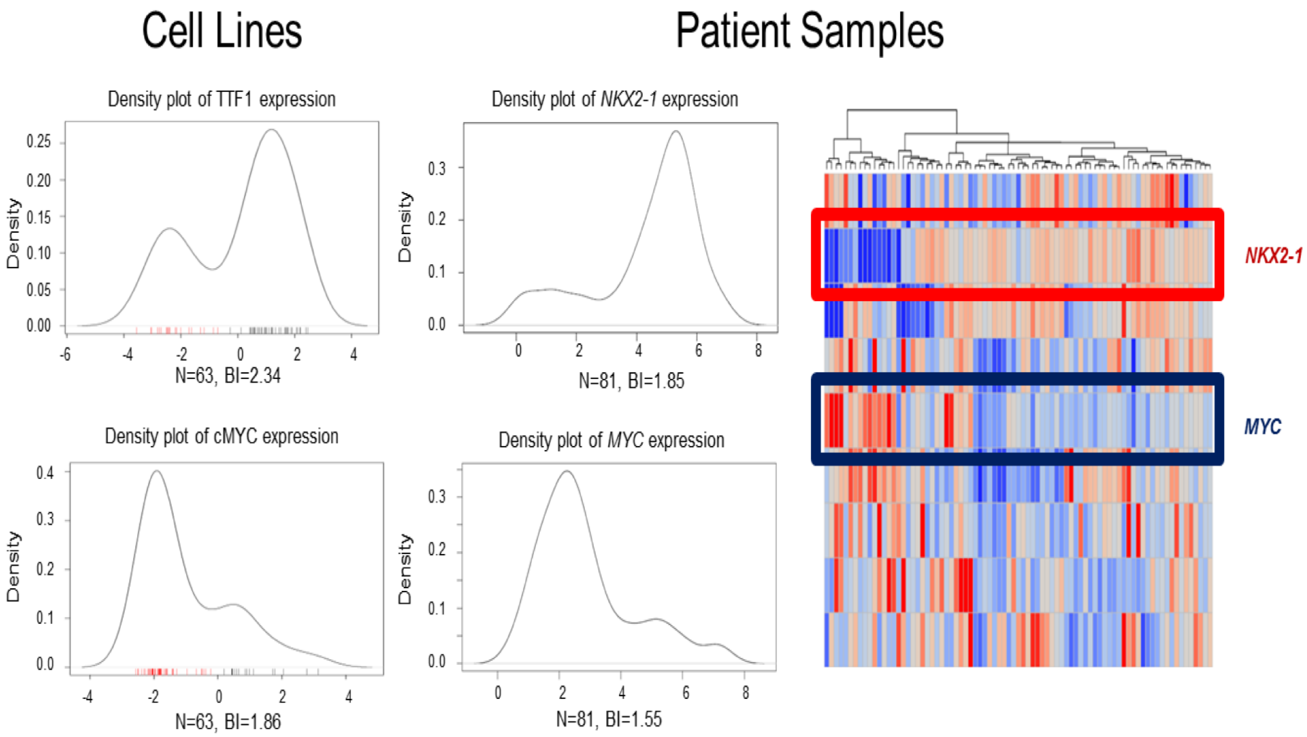

Supplementary Figure 7: These genes were bimodally distributed in both SCLC cell lines (bimodal index (BI) 2.34 and 1.86, for TTF1 and cMYC respectively) and patient tumors.

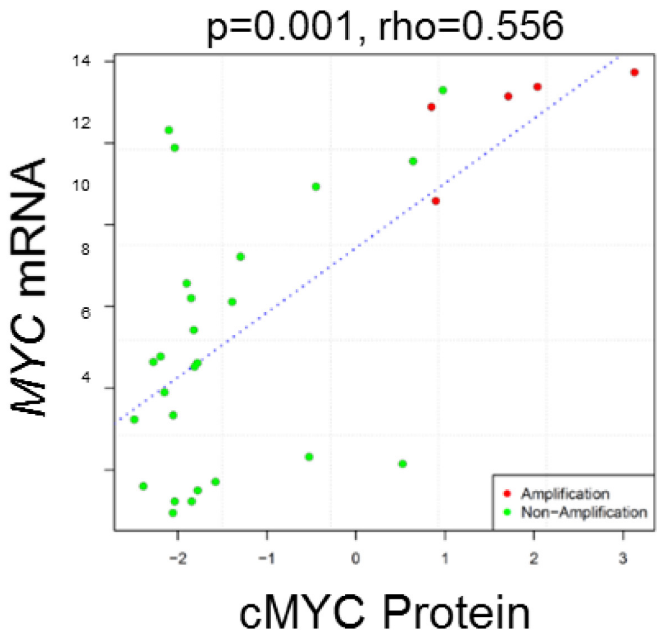

Supplementary Figure 8: cMYC over-expressing cell lines carry a known MYC amplification.

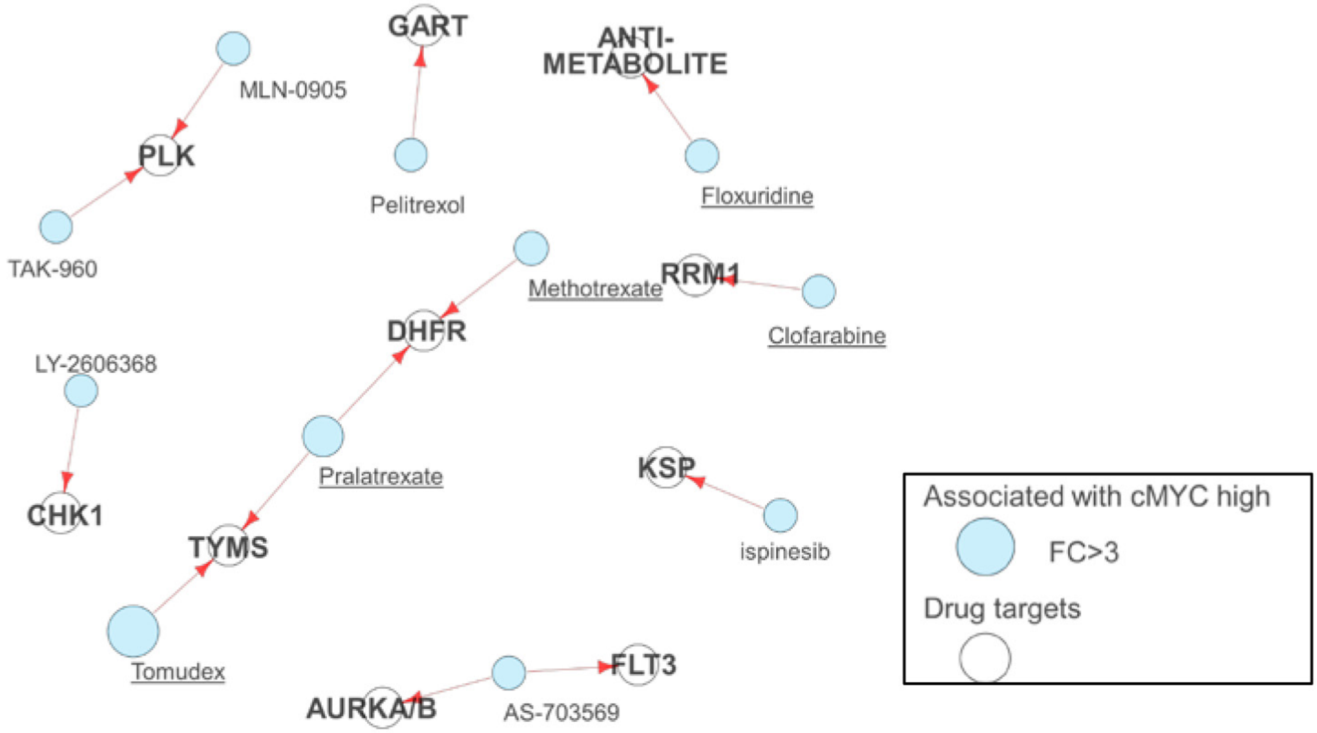

Supplementary Figure 9 : DTECT maps of drugs differentially sensitive between cMYC high and low cell lines (fc>3.0, *p*<0.01).
